# Effects of Subanesthetic Ketamine Administration on Visual and Auditory Event-Related Potentials (ERP) in Humans: A Systematic Review

**DOI:** 10.3389/fnbeh.2018.00070

**Published:** 2018-04-16

**Authors:** André Schwertner, Maxciel Zortea, Felipe V. Torres, Wolnei Caumo

**Affiliations:** ^1^Post-graduation Program in Medicine: Medical Sciences, Federal University of Rio Grande do Sul, Porto Alegre, Brazil; ^2^Laboratory of Pain & Neuromodulation, Clinical Hospital of Porto Alegre, Porto Alegre, Brazil

**Keywords:** ERPs, ketamine, P300, oddball task, cognitive processing

## Abstract

Ketamine is a non-competitive N-Methyl-D-Aspartate (NMDA) receptor antagonist whose effect in subanesthetic doses has been studied for chronic pain and mood disorders treatment. It has been proposed that ketamine could change the perception of nociceptive stimuli by modulating the cortical connectivity and altering the top-down mechanisms that control conscious pain perception. As this is a strictly central effect, it would be relevant to provide fresh insight into ketamine's effect on cortical response to external stimuli. Event-related potentials (ERPs) reflect the combined synchronic activity of postsynaptic potentials of many cortical pyramidal neurons similarly oriented, being a well-established technique to study cortical responses to sensory input. Therefore, the aim of this study was to examine the current evidence of subanesthetic ketamine doses on patterns of cortical activity based on ERPs in healthy subjects. To answer the question whether ERPs could be potential markers of the cortical effects of ketamine, we conducted a systematic review of ketamine's effect on ERPs after single and repeated doses. We have searched PubMed, EMBASE and Cochrane Databases and pre-selected 141 articles, 18 of which met the inclusion criteria. Our findings suggest that after ketamine administration some ERP parameters are reduced (reduced N2, P2, and P3 amplitudes, PN and MMN) while others remain stable or are even increased (P50 reduction, PPI, P1, and N1 amplitudes). The current understanding of these effects is that ketamine alters the perceived contrast between distinct visual and auditory stimuli. The analgesic effect of ketamine might also be influenced by a decreased affective discrimination of sensorial information, a finding from studies using ketamine as a model for schizophrenia, but that can give an important hint not only for the treatment of mood disorders, but also to treat pain and ketamine abuse.

## Introduction

Ketamine is an antagonist of the N-methyl-D-aspartate (NMDA) receptor. It was first synthesized in 1962 and approved by the FDA as an induction agent of general anesthesia in 1970 (Domino, 2010). Ketamine is a dissociative anesthetic with hallucinogenic potential. Nowadays its use in subanesthetic doses (0.3 mg/kg or less) have been explored considerably, mainly as an adjuvant therapy to treat both postoperative acute and chronic pain (Noppers et al., 2010; Michelet et al., 2017). Ketamine has also been used to mimic symptoms of schizophrenia in mechanistic studies with healthy subjects (Jeon and Polich, 2003) and in the treatment of refractory major depression (Mathew et al., 2012). Additionally, the misuse of ketamine as a recreational drug has remarkably increased over the last decade (Degenhardt et al., 2005; Kalsi et al., 2011).

Although interest in ketamine's effects has increased in different settings, its role in cortical neural networks is still poorly understood. Investigations of the neurophysiological mechanisms of ketamine behind its cortical effects could give valuable insights about the consequences of glutamatergic dysfunction, as well as to the assessment of ketamine as a novel treatment for chronic pain and psychiatric disorders. Even though the effects of ketamine at the cortical level have been evaluated in studies using neuroimaging methods, such as functional magnetic resonance imaging (fMRI) and PET (Positron Emission Tomography), it has sometimes been difficult to interpret the effect of ketamine based in these surrogate outcome measures. A possible factor involved in the incongruence across studies are intrinsic properties of these image exams since they only provide an indirect measure of postsynaptic brain activity based on the blood oxygenation level-dependent (BOLD) oscillations. Additionally, it is known that ketamine has a direct vascular effect independent of the NMDA receptor (Noh et al., 2009).

On the other hand, the use of neurophysiological measures like the electroencephalogram (EEG) allow for the direct measuring of electrical brain activity with high temporal resolution. Additionally, the EEG recordings are related to structural and functional components of cognitive processing, thus providing a safe and non-invasive approach to the study of the psychophysiological correlates of cognitive processes. EEG is generally recorded as spontaneous electrical activity or as evoked (event related) potentials (ERPs). ERPs reflect the sequential and parallel activation and synchronization of neural networks in response to an external phasic stimulus, providing quantitative information about ketamine's central impact. According to the type of stimulus or response that is made, ERPs present a typical waveform (Hillyard, 1993). In assessing these measures, we need to consider the shape, the latency and the amplitude of a peak (positive or negative, as defined by the polarity of going voltage). Overall, deflections that occur early, usually before 100 ms, are associated with pre-attentive processes, while the positive peak that can be found after about 300 ms (i.e., P300) is commonly related to attentional processes. Peaks which appear after 300 ms are generally associated with processes of cognitive evaluation. In general, ERPs are consistent within subjects even over several years (Segalowitz and Barnes, 1993).

Studies assessing the effect of ketamine using ERPs have increased in the last decade. Notably, they include a model with healthy subjects that use ketamine to induce behavioral and electrophysiological effects that mimic findings observed in schizophrenic patients (Jeon and Polich, 2003). A reduction of ERP-amplitudes across different task conditions, as oddball P300 (Oranje et al., 2000, 2009; Watson et al., 2009) and mismatch negativity (MMN) (Umbricht et al., 2002; Rosburg and Kreitschmann-Andermahr, 2016), was observed upon subanesthetic ketamine challenge. These findings have been reported mostly without exception, and similar changes have been seen in schizophrenic patients (Turetsky et al., 2007; Javitt et al., 2008). We can infer that the reversing of these ERP changes by pharmacological treatment may indicate an improvement in cognitive functions.

Given the emerging importance of ketamine as a potential novel treatment for mood and pain chronic conditions and as a drug of abuse, we have reviewed the current knowledge of ketamine's effect in ERPs and discussed how these finding can impact the comprehension of its cortical effects.

## Methodology of the literature review

This systematic review was reported in accordance with the PRISMA (Preferred Reporting Items for Systematic Reviews and Meta-Analyses) guideline. There was no pre-published protocol.

### Search strategy

To identify relevant studies, a literature search was conducted in MEDLINE (from 1966), ScienceDirect (from 2006), EMBASE and Cochrane Databases (from 1993) using the following keywords: “ERP” (or “event related potentials”) and “ketamine” up to Dec 20th, 2017. In studies detected by this search, the reference lists were checked for additional unidentified studies. In order to obtain a maximum number of studies, reviews focusing on electrophysiological effects of ketamine were also assessed.

### Study selection: inclusion and exclusion criteria

In our analysis, we included only randomized double blind controlled studies that used either ketamine or S-ketamine as a psychoactive drugs in healthy participants and recorded any ERP component by electroencephalographic recordings. S-ketamine exhibits a blockade of NMDA receptors twice as large as R-ketamine and larger analgesic efficacy (Arendt-Nielsen et al., 1996). Studies using S-ketamine were included considering its current interest and use in the pain management setting. There was no language or date restriction. The manuscripts with the following criteria were excluded: (1) animal studies, (2) studies which lacked a control group or baseline condition, (3) studies that did not report original data, and (4) *in vitro* studies.

The studies retrieved using the search strategy were screened independently by two review authors by evaluation of titles and abstracts to identify duplicates and select studies that potentially meet the inclusion criteria. After initial assessment, the full texts of the potentially eligible studies were evaluated for eligibility independently by two review team members. Disagreements over the eligibility of particular studies were discussed with a third reviewer. Data of included studies was extracted using a standardized form for evidence synthesis and assessment of study quality. Information retrieved from eligible studies included sample sizes, gender proportion, ketamine doses, cognitive assessments performed, type of analysis, significant findings, and their effect sizes.

Due to the varying methods, the studies were initially separated into two groups according to the studied task: visual ERP studies and auditory ERP studies. Next, studies were grouped according to the investigated ERP component into seven subgroups: (1) P300, (2) sensory gating, (3) N100 and P100, (4) N170, (5) N200 and P200, (6) processing negativity, and (7) mismatch negativity.

When possible, effect sizes were calculated for significant findings. In instances where the necessary data was unavailable, best efforts were made to contact the author. For all findings with available data, effect sizes were reported and also converted to Cohen's d for comparable results across studies.

### Risk of bias assessment

In order to evaluate and describe the quality of the studies, two review authors independently assessed the risk of bias in included studies according to the Grading of Recommendations Assessment, Development and Evaluation (GRADE) methodology for clinical trials (Farrell et al., 2016), by considering the following characteristics: random sequence generation (selection bias), allocation concealment (selection bias), blinding of participants and personnel (performance bias), blinding of outcome assessment (detection bias), incomplete outcome data (attrition bias), selective reporting (reporting bias); and other biases. Green plus signs mean the bias risk is low and methodology was well-described, blank spaces mean the risk was not reported or impossible to evaluate due to lack of information and red minus signs mean high risk of bias (**Table 2**). Disagreements between the review authors over the risk of bias in particular studies were resolved by discussion, with the involvement of a third review author where necessary.

## Results

**Figure 1:**
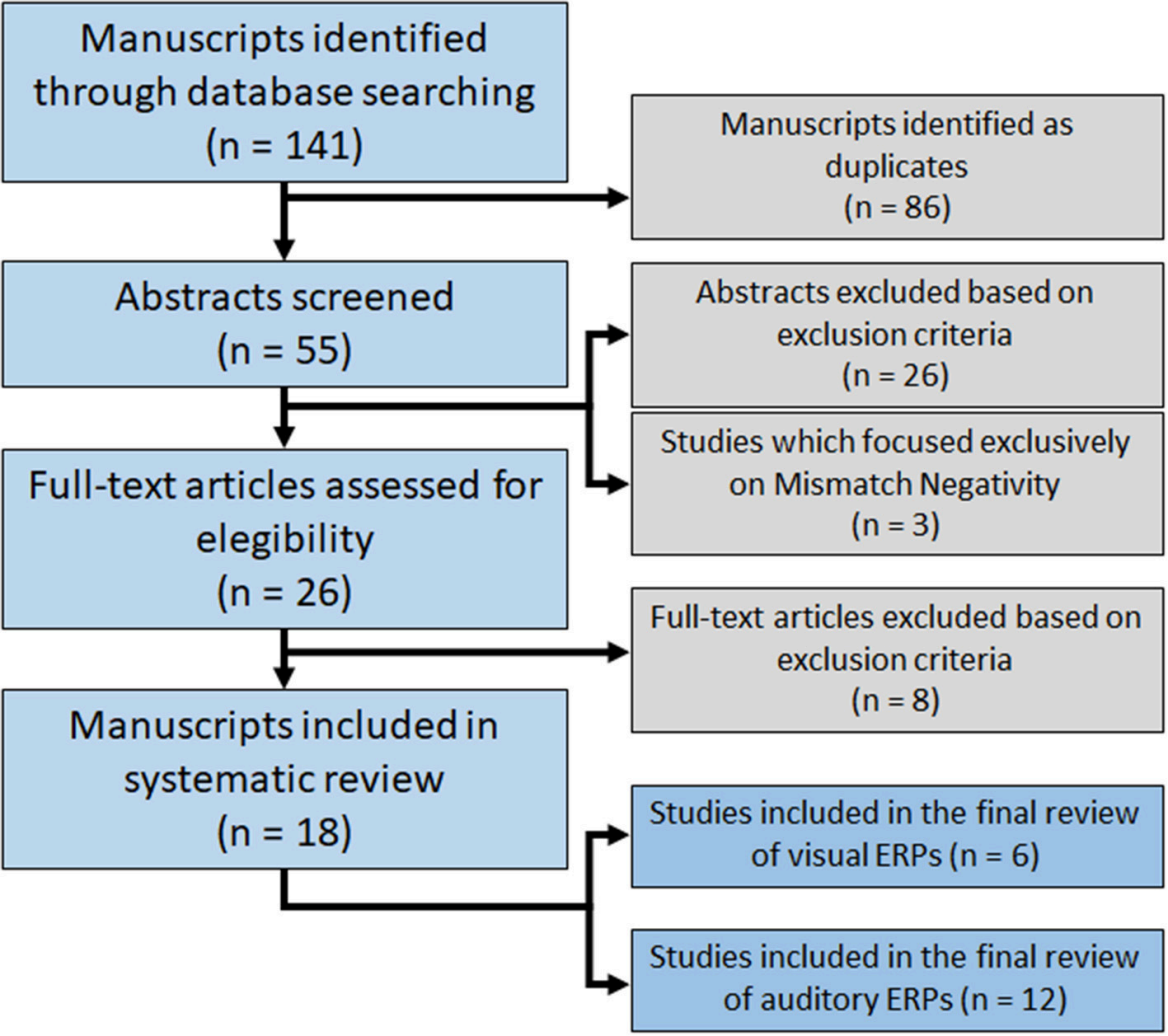
Flowchart of database searching for relevant studies.

A total of 141 potentially relevant articles were identified. By title evaluation, duplicates were removed and the abstracts were then read to identify manuscripts that described studies looking at the effects of ketamine on ERPs and met the inclusion and exclusion criteria. Of the 26 articles identified through database searches, 18 were included in this systematic review (Figure 1). Articles are summarized in Table 1.

**Table 1:** Details of the studies included in the current systematic review.

**Authors and year**	**Participants (male, female)**	**Cognitive Tasks: stimuli**	**ERP Outcomes**	**Electrode**	**Significant results**	**Effect size (η^2^)**	**Cohen's d**
**VISUAL ERP STUDIES**							
Ahn et al., 2003	10 healthy subjects (10 M, 0 F)	Sternberg's short-term memory scanning task: 5–7 digits memory task	P300 amplitude and latency	Fz, Cz, and Pz	↓ P300 amplitude no drug effect on latency	0.43 –	0.97 –
Watson et al., 2009	23 healthy subjects (15 M, 8 F)	Visual oddball task: standard (small blue circle, white background, 80%), target (large blue circle, white background, 10%), and novel (non-repeating fractal images, 10%)	P3a and P3b amplitude and latencies	Pz	↓ P3a and P3b amplitudes no effect on P3 latency no effects on N200 t and P200t	0.76 – –	2.41 – –
			N200 and P200 components elicited by both targets and novels	Fz	↑ P3a amplitude	0.34	0.16
					↓ P3a latency	0.74	2.23
					↓ N200 amplitude (target and novels)	0.89	5.82
					↑ P200 amplitude (novels)	0.44	1.00
Knott et al., 2011	40 healthy subjects (20 M, 20 F); half smokers, half non-smokers	Visual information processing task: single digits (1–9) presented (black on white) in the center of a monitor at a fixed rate of 110 digits/min,	P300 amplitude	Pz	↓ P300 amplitude no effect on latency	0.71	1.07
Musso et al., 2011	24 healthy subjects (24 M, 0 F)	Visual oddball task: black and white checkerboard reversal consisting of 64 target and 256 non-target stimuli	P300 amplitude and latency	Pz	↓ P300 amplitudes no effect on latency	0.53 –	1.27 –
Schmidt et al., 2013	21 healthy subjects (12 M, 9 F)	Backward masking paradigms (facial affect discrimination); fearful vs. neutral faces and happy vs. neutral faces	P100	P08/P8/P10/O2 and PO7/P7/P9/O1	no drug main effect	–	–
			N170		↓ N170 amplitudes to emotional faces no effect on latency	0.30	0.93
Koychev et al., 2017	44 healthy subjects	Delayed matching-to-sample WM task using abstract forms: participants were instructed to remember one, two, or three abstract forms presented successively. After a delay period, a new or previously presented form appeared on the screen, and the participants pressed a button indicating if they did or did not recognize the form from the sample	P100	Averaged occipital (PO8, O2, O1, PO7, and Oz)	↑ P100 amplitude no effect on latency	0.12	0.74
			P300	Averaged parietal (P1, Pz, and P2)	↓ P300 amplitude in a load-dependent manner no effect on latency	0.10	0.67
**AUDITORY ERP STUDIES**							
van Berckel et al., 1998	18 healthy subjects (18 M, 0 F)	Blocks of 36 click pairs with an interstimulus interval of 500 ms, and an intertrial interval of 10 s	PPI of the startle reflex		no drug effect	–	–
			P50	Cz	no drug effect	–	–
Umbricht et al., 2000	20 healthy subjects (14 M, 6 F)	Auditory MMN task: standard (1,000 Hz) 100 ms duration, deviant pitch tones (1,500 Hz) 100 ms duration, and deviant duration tones, 250 ms duration;	N1		↑ N1 amplitude; no effect on latency	0.41	0.20
			P2		no effect on amplitude and latency	–	–
Oranje et al., 2000	18 healthy subjects (18 M, 0 F)	Auditory selective attention task: standard (1,000 Hz) and target tones (1,100 Hz), 50 ms duration	N100	Cz	↑ N100 amplitude to deviant stimuli; no effect on N100 latency	–	–
			P300	Pz	↓ P300 amplitude (both in general and to deviant stimuli in particular) no effect on latency	–	–
			PN	Fz	↓ PN	–	–
Kreitschmann-Andermahr et al., 2001	13 healthy subjects (12 M, 1 F)	Auditory MMN task: standard (1,000 Hz) 50 ms duration, deviant pitch tones (1,050 Hz) 50 ms duration, and deviant duration tones (,1000 Hz), 100 ms duration;	N100 (as secondary outcome)	Fz	no drug effect	–	–
Umbricht et al., 2002	20 healthy subjects (14 M, 6 F)	Auditory MMN task: standard (1,000 Hz) 100 ms duration, deviant pitch tones (1,500 Hz) 100 ms duration, and deviant duration tones, 250 ms duration;	N100	Fz	no drug effect	–	–
			P200	Fz	no drug effect	–	–
Oranje et al., 2002	18 healthy subjects (18 M, 0 F)	Auditory selective attention task: standard (1,000 Hz) and target tones (1,100 Hz), 50 ms duration	PPI		no drug effect	–	–
			P50		no drug effect	–	–
Murck et al., 2006	16 healthy subjects (8 M, 8 F)	Auditory sensory gating paradigm: 1,000 Hz 100 ms duration in a pseudorandom sequence; interstimulus intervals ranging from 4 to 8 sec	N100-P200 peak-to-peak amplitude	Cz	↓ N100-P200 peak-to-peak amplitude no effect on latency	0.78	2.55
Heekeren et al., 2007	15 healthy subjects (9 M, 6 F)	Auditory MMN task: standard (1,000 Hz) 50 ms duration, deviant pitch tones (1,200 Hz) 50 ms duration, and deviant duration tones (1,000 Hz), 100 ms duration;	N100 (as secondary outcome)	Fz	no drug effect	–	–
Oranje et al., 2009	18 healthy subjects (18 M, 0 F)	Auditory selective attention task: standard (1,000 Hz) and target tones (1,100 Hz), 50 ms duration	P300 amplitude	Pz	↓ P300 amplitude, more pronounced for deviant than for standard stimuli, irrespective of attention no effect on latency	0.54	1.28
			PN	Fz	↓ PN	0.72	2.11
Gunduz-Bruce et al., 2012	16 healthy subjects (13 M, 3 F)	Auditory oddball (P300) paradigm: 150 stimuli comprising 120 standards (80%), 15 targets (10%), and 15 novels (10%)	P300	Pz Fz	↓ target P3b amplitude no effect on latency ↓ novelty P3a amplitude no effect on latency	0.85 0.24	3.36 0.49
Mathalon et al., 2014	8 healthy subjects (5 M, 3 F)	Auditory oddball target detection task: standard (500 Hz), target (1,000 Hz) 50 ms duration, and novel distractor sounds, (average duration of 250 ms)	P3b P3a	Cz, Pz and Fz	↓ P300 amplitude (P3a and P3b) [in Cz, but not in Fz and Pz] ↑ P300 latency (P3a and P3b)	0.61	1.57
						0.87	3.56
Kort et al., 2017	31 healthy subjects (19 M, 12 F)	Talk and listen task: vowel “a”	N1 suppression during vocalization	Fz, Cz	↓ N1 suppression during talk compared to listening	0.34	1.45

*PN, processing negativity; MMN, mismatch negativity; WM, working memory*.

Six studies used visual stimuli to generate ERPs, while 15 used auditory stimuli. Considering the recent meta-analysis regarding the effects of ketamine on MMN, three papers that exclusively studied MMN were not reviewed in detail here (Roser et al., 2011; Schmidt et al., 2012; Hamilton et al., 2018). Therefore, 12 studies were included in the final review of auditory ERPs.

The 18 studies included in this systematic review collected data in 373 (291 male, 82 female) healthy participants. The used ketamine dosages used and plasmatic levels showed some variance between studies (Table 2). Bias was strongly suspected in one study (Kreitschmann-Andermahr et al., 2001). It was categorized high-risk of bias in randomization, blinding and “other risk of bias”. Most studies did not clearly describe the methodology used to compute the sample size for the primary outcome. Most studies categorized as high-risk in “other risk of bias” used models that did not achieve stable ketamine plasmatic levels. Although drug administration was usually double-blind, the psychomimetic effects of ketamine compromise complete blinding for both participants and personnel.

**Table 2:** Ketamine administration and risk of bias.

**Authors and year**	**Substance**	**Bolus (mg/kg)**	**Continuous injection (mg/kg per hour)**	**Plasmatic level range (ng/ml)**	**Risk of bias**						
					**(A)**	**(B)**	**(C)**	**(D)**	**(E)**	**(F)**	**(G)**
Ahn et al., 2003	Ketamine	0.26	0.65	–			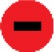		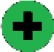	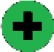	
Watson et al., 2009	Ketamine	0.23	0.58	–			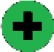			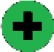	
Knott et al., 2011	Ketamine	0.04	–	–			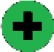				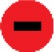
Musso et al., 2011	S-ketamine	0.10	0.9375^a^	–						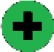	
Schmidt et al., 2013	S-ketamine	0.14^b^	0.36^a^	–	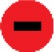						
Koychev et al., 2017	Ketamine	0.16	0.39	100^c^				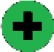			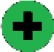
van Berckel et al., 1998	Ketamine	0.30	0.213	129–158					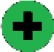	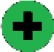	
Umbricht et al., 2000	Ketamine	0.24	0.9	–				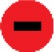	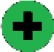	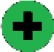	
Oranje et al., 2000	Ketamine	0.30	0.213	129–158				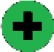	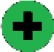		
Kreitschmann-Andermahr et al., 2001	Ketamine	0.30	–	82–426	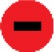	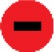	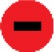	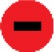			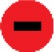
Umbricht et al., 2002	Ketamine	0.24	0.9	–				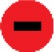	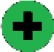	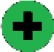	
Oranje et al., 2002	Ketamine	0.30	0.213	116–122				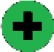	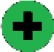		
Murck et al., 2006	S-ketamine	–	0.056^b^								
Oranje et al., 2009	Ketamine	0.30	0.213	116–122		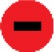	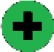			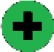	
Heekeren et al., 2007	S-ketamine	0.15–0.2	0.6–0.9^a^	–				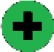		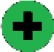	
Gunduz-Bruce et al., 2012	Ketamine	0.23	0.58	66–75						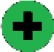	
Mathalon et al., 2014	Ketamine	0.26	0.65	174–222					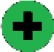	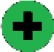	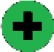
Kort et al., 2017	Ketamine	0.23	0.58	–					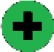	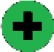	

a The dose was further reduced by 10% every 10 min;

b Average dose considering a mean body weight of 70 kg;

c Estimated value based on a pharmacological model;

*Risk of bias: (A) Random sequence generation (selection bias), (B) Allocation concealment (selection bias), (C) Blinding of participants and personnel (performance bias), (D) Blinding of outcome assessment (detection bias), (E) Incomplete outcome data (attrition bias), (F) Selective reporting (reporting bias), (G) Other bias (did not achieve stable ketamine plasmatic level). Green plus signs mean the bias risk is low and methodology was well described, blank spaces mean the risk was not reported or impossible to evaluate due to lack of information and red minus signs mean high risk of bias*.

## Discussion

In this systematic review, we compiled data regarding ketamine's effect assessed by ERP signals as a way to understand its potential cortical electrophysiological effect in experimental studies with healthy individuals. The ERPs as an outcome were used as a measure to capture neural activity related to both visual and auditory processes. We attempt to present an overview of the different ERP components and the main findings in a set of different experimental conditions involving ketamine administration. The waveforms are described according to latency and amplitude. ERPs in humans can be classified in two categories: (1) waves that peak roughly within the first 100 ms after stimulus, classified as early components, which depends mainly on the physical parameters of the stimulus, and (2) the later components that reflect information processing, corresponding to the subject evaluation of the stimulus are termed “cognitive” or “endogenous.” The discussion is presented according to ERP by millisecond as a record of neural information processing.

According to our evaluation, most of the studies reached good methodological quality, indicating the reported results are quite reliable (Table 2). It was specially observed for reports on P300 complex (Watson et al., 2009; Mathalon et al., 2014), P100 (Koychev et al., 2017), N100 (Oranje et al., 2000, 2002; Kort et al., 2017), and PPI (van Berckel et al., 1998; Heekeren et al., 2007). The study of Kreitschmann-Andermahr et al. (2001) could be ranked as having the poorest methodological quality among the included studies, although they report the same null effects of ketamine as Heekeren et al. (2007) using a very similar cognitive task.

### P300

P300 (or P3) is the most widely known and studied ERP. It was first described over 35 years ago and since then it has provided fundamental information on the neural basis of normal and dysfunctional cognition (Sutton et al., 1965; Bashore and van der Molen, 1991). P300 is frequently elicited using variations of the “oddball” paradigm where two sensorial stimuli are presented with different probabilities (most frequently 80:20) in a random order. In this task, the subject is required to discriminate the infrequent target stimulus from the frequent standard stimulus. Typically, the individual is instructed to note the occurrence of target stimulus by pressing a button or mentally counting. The ERP elicited by the target stimulus is a positive-going potential with a peak latency of about 300–350 ms for auditory stimuli and 350–450 ms for visual stimuli with a larger amplitude over the parietal electrode sites (Johnson, 1988; Picton, 1992).

The major theoretical interpretation is that the P300 amplitude indexes neural processes stemming from “tasks that are required in the maintenance of working memory” (Fabiani et al., 1986), and update of the mental model of the stimulus environment (Coles et al., 1988; Polich and Donchin, 1988). Also, the P300 amplitude is proportional to the attentional resources employed in a given task (Kramer et al., 1988), and the magnitude of the component has been associated with memory performance (Fabiani et al., 1986). Therefore, the P300 amplitude can be viewed as a measure of central nervous system activity that occurs in the generation of stimulus memory representations, and the size of the component reflects the degree to which information is processed (Polich and Comerchero, 2003). The P300, despite its simplicity, provides important information about the brain activity underlying some fundamental cognitive operations.

Interest in the investigation of ERP with ketamine administration increased in the last few years after it was demonstrated that ketamine-induced electrophysiological effects in healthy subjects correspond to findings in schizophrenic patients (see Jeon and Polich, 2003 for a review). The effects of ketamine on P300 were studied using varied tasks and contexts. Visual evoked P300s were evaluated using different kinds of stimulation, such as concrete geometrical forms (Watson et al., 2009), black and white checkerboard reversals (Musso et al., 2011), digits (Ahn et al., 2003; Knott et al., 2011), and abstract forms (Koychev et al., 2017). However, the result is similar, regardless of the stimulus category: ketamine consistently attenuated parietal P300 amplitudes without changing P300 latencies.

Similar results regarding auditory stimulation were reported by at least three groups. They report that ketamine attenuates auditory P300 amplitudes (Oranje et al., 2000, 2009; Gunduz-Bruce et al., 2012; Mathalon et al., 2014) with no effects on latency. Only one study found prolonged P300 latencies using lower frequency tones (standard 500 Hz, target 1,000 Hz) (Mathalon et al., 2014).

#### Novelty processing: P3a e P3b

The classic oddball task was further modified to a 3-stimulus paradigm in which a third infrequent, non-target-stimuli was inserted into the sequence of target and standard stimuli. When novel stimuli (e.g., dog barks, color forms, etc.) are presented as infrequent non-target stimuli in the series of more typical target and standard stimuli (e.g., tones, letters of the alphabet, etc.), a larger P300 is produced over the frontal/central electrodes with auditory, visual, and somatosensory stimuli (Courchesne et al., 1984; Knight, 1984; Yamaguchi and Knight, 1991). This novelty P300 component is sometimes called the “P3a,” whereas the parietal maximum P300 from the target stimulus is sometimes called the “P3b” (Comerchero and Polich, 1999). Therefore, studies that do not differentiate between P300 components usually evaluate the ERP generated from target stimuli (which, in the last analysis, corresponds to P3b).

The (target) P3b (elicited by infrequent, task-relevant target stimuli) is commonly associated with voluntary attention and the updating of working memory (Coles et al., 1988), while the (novelty) P3a (elicited by infrequent, task-irrelevant stimuli) likely indexes an automatic orienting response to novel stimuli. Although ketamine affects ERPs elicited by targets (P3b), its most striking effects were on the ERP correlates of novelty processing. Previous reports in both human and animal literature describe the involvement of the NMDA neurotransmission in the processing of novel stimuli (Grunwald and Kurthen, 2006). Investigations of the effects of ketamine in a 3-stimulus paradigm found controversial alterations in P3a topography and latency (Watson et al., 2009).

Specifically, ketamine produced a small increase in P3a amplitude in frontal sites, along with a decrease in P3a latency relative to placebo in a visual oddball paradigm (Watson et al., 2009). However, studies that used auditory stimulation found a decrease in frontal P3a amplitudes in a way that was similar to parietal P3b amplitudes (Gunduz-Bruce et al., 2012). One study also reported increased latency for P3a (Mathalon et al., 2014).

Most studies exploring the effects of ketamine on ERPs were conducted to evaluate ketamine as a model for schizophrenia, so the results of lower P300 (P3b) amplitudes were in line with previous findings in schizophrenic patients. However, interpretation of these findings must take into consideration that target-elicited ERPs during oddball tasks involve widespread, and in part simultaneous, cortical activation. It is important to point out that generators of the P300 recorded at scalp are not so well-defined due to inter-subject variability, and therefore, the reduction in P300-amplitudes induced by ketamine does not necessarily reflect “hypoactivation” of cortical areas but can also indicate a “ceiling effect” secondary to increased cortical baseline activation.

The P3a latencies are often interpreted as a marker of the speed of stimulus processing and classification in healthy adults. It is therefore reasonable to speculate that P3a latency changes following ketamine could indicate that it alters the speed with which novel stimuli are detected and processed. It's not clear if the different results using visual and auditory stimulation reflect specific actions of ketamine over visual and auditory cortices or if they are secondary to inherent differences of visual and auditory information processing.

### P2 and N2

The P2 and N2 components have also been evaluated in the context of novelty processing. The N2(00) is usually interpreted as an index of response inhibition (Falkenstein, 2006) and/or conflict monitoring (Donkers and van Boxtel, 2004). It is mediated by the anterior cingulate cortex and other prefrontal regions (Wang et al., 2003; Bertoli and Probst, 2005). The P2(00) has been associated with avoidance of invalid behavioral responses, suppression of irrelevant stimulus features to improve performance (Potts, 2004), or the effortful allocation of attention in a variety of tasks (Crowley and Colrain, 2004; Falkenstein, 2006).

Ketamine was found to decrease N2 while increasing P2 amplitudes to novel stimuli in a visual paradigm (Watson et al., 2009) but did not affect P2 amplitudes in an auditory paradigm (Umbricht et al., 2000). As part of the normal response to visual stimuli, the P2 has been shown to be enhanced by feature-based attention (Luck and Hillyard, 1994). Accordingly, the effects of ketamine on visual P2 and N2 amplitudes might be the result of an impairment of ACC activity related to detection and processing of infrequent stimuli. NMDA antagonism appears to alter the efficiency of early attentional processes indexed by these two components, affecting multiple cognitive operations related to novelty processing. If ketamine alters the perceived salience of the novel stimuli, this could necessitate changes in the cognitive resources required to avoid inappropriate responses.

### Sensory gating

The term “sensory gating” describes a group of neurological processes responsible for filtering out redundant stimuli, thus preventing an overload of unnecessary information in the higher cortical centers. The P50 reduction has been most commonly used in the setting of electrophysiological studies to evaluate this early processing of sensorial stimuli.

The P50 is a pre-attentional positive ERP peak, which appears about 50 ms after the onset of a stimulus. When two identical auditory stimuli are presented, one is the conditioning stimulus while the other is the test stimulus. If they are presented with an inter-stimulus interval of 200–2,000 ms, the P50 component evoked by the second stimulus is usually smaller than the P50 evoked by the first one (P50 reduction).

The prepulse inhibition of the startle reflex (PPI) is another model (not involving scalp EEG recordings) frequently used along with P50 reduction to evaluate sensory gating. It consists in the evaluation of the startle reflex, usually the evaluation of the eye blink reflex amplitude and latency (measured with electromyography) in response to a strong acoustic stimulus. When this sound is preceded by a weak stimulus, the amplitude and latency of the reflex is reduced.

Ketamine was expected to reduce both P50 and PPI, as they have been reported to be decreased in schizophrenic patients when compared to healthy subjects (van Berckel et al., 1998). However, studies of ketamine's effects on PPI and P50 reduction have not found evidence of a decrease in sensory gating, even though ketamine induced several psychomimetic effects, analgesia and coordination problems (van Berckel et al., 1998; Oranje et al., 2000). Rather, in some stimulus conditions and in higher doses (0.23 and 0.5 mg/kg), ketamine increased PPI (Duncan et al., 2001; Abel et al., 2003).

Along with P50, N100 and P200 amplitudes have also been described to be reduced in schizophrenic patients (Shelley et al., 1999). Murck et al. (2006) found that ketamine decreased N100-P200 peak-to-peak amplitudes after a sensory gating auditory paradigm, even using lower ketamine doses (0.05 mg/kg/h) (Murck et al., 2006). The different ketamine doses, variation in timing of sensory gating testing and the possible confounding variable of smoking status are likely to explain the difference between findings. Therefore, in humans, perhaps due to methodological problems, it is not clear if ketamine decreases sensory gating in a similar way to the impaired gating observed in schizophrenia.

### Early components: P100 and N100

The N100 (or N1) is a negative evoked potential occurring at around 100 ms after the onset of a stimulus, while P100 (or P1) is a correspondent positive ERP occurring in the same window of time. Both N100 and P100 reflect obligatory, exogenous sensory responses, and their amplitudes are influenced by several factors, including interstimulus interval, stimulus intensity, arousal level, and subjects' attention (Hillyard, 1993; Luck et al., 2000). They are supposed to reflect the early evaluation of the emotion or salience of the stimulus that occurs before more complex perceptual analyses are completed (Vuilleumier and Pourtois, 2007). In particular, lateral occipital P1, anterior and posterior N1 effects have been thought to reflect top-down gain-control of the initial feed-forward sensory activity and stimuli discrimination, respectively (Leonard et al., 2013).

Only three studies have assessed the effects of ketamine on N100 and P100. A recent study has shown that ketamine increased visual P100 potentials during encoding and retrieval phases of a working memory task (Koychev et al., 2017), whereas another study did not find any effect of ketamine on P100 in a visual emotional-face recognition task (Schmidt et al., 2013). However, a prior study reported that ketamine increased N100 amplitudes in an auditory paradigm (Umbricht et al., 2000). These findings went against the prediction that the pattern of decreased P100 and N100 amplitudes observed in schizophrenia would be replicated.

The P1 component is an early exogenous sensory potential generated in the extrastriate visual cortex (Clarke, 1994). Larger P1 amplitudes have been seen during perception of “attention-grabbing” stimuli such as fearful vs. neutral faces (Pourtois et al., 2005), reward-associated visual features (Hickey et al., 2010), or stimuli appearing in a validly cued location (Hillyard et al., 1998). Larger P1s in these conditions have been suggested to reflect attention modulation on sensory gain that aids early visual processing (Hillyard et al., 1998). PET metabolic mapping studies demonstrated that ketamine focally increases prefrontal cortex metabolism (glucose uptake). They hypothesized that this response might be related to the disinhibition of local glutamate release (Breier et al., 1997; Holcomb et al., 2001). Other studies using fMRI indicate that ketamine causes complex regional blood oxygen level-dependent (BOLD) changes, especially in prefrontal and parietal cortices, including hypo and hyperactivation (Deakin et al., 2008).

Thus, early sensory processing changes (reflected by N1 and P1 increased amplitudes) may be caused, at least in part, by a loss of top-down control related to prefrontal dysfunction induced by ketamine. A noisier signal and disinhibition of long-range facilitatory projections to visual and auditory cortices from the prefrontal cortex could thus account for the increased P1 and N1 amplitudes.

Also, another complementary explanation for these results is that the ketamine-induced visual P100 increases could be secondary to loss of the NMDA modulation of sensorial information and a potentiation of feedforward alpha-amino-3-hydroxy-5-methyl-4-isoxazole propionic acid (AMPA) mediated processes. In line with this idea is the observation that under ketamine participants report heightened perceptual experiences (Lahti et al., 2001). The NMDA hypofunction might disrupt the excitatory (glutamate) and inhibitory (gamma-aminobutyric acid) balance in the neural circuitry (Krystal et al., 2003; Anticevic et al., 2013). Considering that NMDA receptors facilitate modulatory feedback in the visual cortex, while AMPA receptors underlie feedforward processes (Self et al., 2012), the blocking of NMDA receptors may lead to gamma-aminobutyric acidergic disinhibition and therefore, to an increase in bottom-up stimulation of AMPA receptors.

This hypothesis is supported by findings in preclinical research, which have shown that the block of NMDA receptors induced by ketamine is associated with a consequent disinhibition of glutamate release and activation of AMPA receptors (Moran et al., 2015). However, at present, any conclusive interpretation regarding the global cortical effects of ketamine is probably an oversimplification. Hence, further studies are needed to allow definitive conclusions.

Recently, a study found that ketamine lead to a suppression of auditory N1 evoked by speech sound during vocalization relative to passive listening (Kort et al., 2017). In the talk/listen task, EEG was obtained as participants said the single vowel “a” and then passively listened to their speech being played back. Under physiological conditions, robust N1 amplitude suppression occurs to self-produced speech but ketamine induced dysfunction in this predictive coding during vocalization. The fact that ketamine specifically altered the N1 response during self-produced vocalizations but not during passive listening to these vocalizations being played back suggest that N1 suppression deficits were not due to changes in sensory perception but to changes in predicting the sensory consequences of talking.

### N170

The N170 is a negative occipitotemporal potential at ~170 ms post-stimulus and has been associated with the structural encoding of facial configurations (Itier and Taylor, 2004; Rossion and Jacques, 2008). The N170 not only reflects face sensitivity but also emotional sensitivity during conscious as well as non-conscious face processing. N170 amplitudes have been correlated with severe depressive symptoms (Noll et al., 2012) and significantly differ between healthy volunteers and patients with bipolar disorder (Degabriele et al., 2011; Sokhadze et al., 2011). Considering these N170 implications to mood disorders, one study evaluated the effect of S-ketamine in a task using emotional (fearful and happy) faces. Ketamine was found to impair the encoding of emotional faces as shown by reduced N170 amplitudes over the parieto-occipital electrode sites. Hence, these findings, along with previous evidence, suggest that the ketamine-induced effect on limbic and visual regions is associated with the emotional blunting and depersonalization phenomena evident in ketamine states (Krystal et al., 1994).

### Processing negativity

Processing negativity (PN) is a negative deflection that appears over frontal cortical areas when subjects are asked to attend selectively to specific stimuli defined by certain characteristics, while ignoring others (e.g., stimulus presented on left/right ear) (Oranje et al., 2009). PN is usually expressed as the difference between the ERPs to the attended and the unattended stimuli. Again, in patients with schizophrenia, EEG recordings showed reduced PN compared to healthy subjects. Only one group studied the effects of ketamine on PN, reporting that ketamine reduced processing negativity in healthy volunteers (Oranje et al., 2000, 2002, 2009).

PN and P300 amplitude represent two different levels of attention. Processing negativity was first described by Hillyard et al. (1973) and Hillyard (1993). Given the early latency of the phenomenon, they suggested that the underlying attentional process is a tonically maintained set favoring one ear over the other, rather than an active process of discrimination or recognition of each individual stimulus separately. In contrast, the P300 amplitude is assumed to reflect aspects of further conscious processing of an attended stimulus.

Taking into account evidence of counteraction of the effects of ketamine on PN by dopaminergic antagonists (Oranje et al., 2009), the current hypothesis is that ketamine's effect on reducing PN is mediated by its dopaminergic (D2) receptor agonism (Kapur and Seeman, 2002; Seeman and Guan, 2008). Thereby, in contrast to P300 amplitudes, processing negativity may involve dopaminergic D2 activity.

### Mismatch negativity

MMN is a negative auditory ERP peaking around 100–150 ms. It follows any discriminable deviant sound occurring during repetition of standard sounds (Näätänen, 1995; Gunduz-Bruce et al., 2012). The MMN is elicited automatically by the deviant sound, even without conscious direction of attention to the auditory channel. The main interpretation of MMN is that it reflects the echoic memory of auditory sensory information, as the cognitive processes for detection of deviance require an online representation of the “standard” stimulus in the auditory stream (Todd et al., 2008).

Considering that a recent review and meta-analysis regarding ketamine effects on MMN was performed (see Rosburg and Kreitschmann-Andermahr, 2016), we will not focus in MMN in the present review and, therefore, papers that studied exclusively MMN were not reviewed in detail here (Roser et al., 2011; Schmidt et al., 2012; Hamilton et al., 2018). The meta-analysis of Rosburg and Kreitschmann-Andermahr (2016) included the studies of Roser et al. (2011) and Schmidt et al. (2012). The study of Hamilton et al. (2018) focused on the interaction of nicotine and ketamine on MMN. Baseline MMN amplitude was found to predict the extent of the ketamine-induced psychotic symptoms, with smaller MMN amplitudes being associated with stronger psychomimetic effects. This fact leads to the hypothesis that MMN amplitudes might index the functional state of the neurotransmission mediated by the NMDA receptor (Umbricht et al., 2002). Current understanding on this topic is that ketamine is able to reproduce an electrophysiological pattern that resembles the MMN deficits found in chronic schizophrenia patients: decreased MMN amplitudes and increased MMN latencies, widely independent of the eliciting deviance used.

### Limitations

As far as we can tell, the number of studies examining the cortical effects of ketamine using ERPs is relatively small. Additionally, considering the wide variation of ketamine administration regimens and the multiplicity of outcomes evaluated (ERPs), no meta-analysis was performed. Most studies are exploratory and present methodological limitations related to sample size, randomization, and blinding. Although drug administration was usually double-blind across studies, the ketamine group was generally apparent given its psychomimetic effects, mainly in crossover designs when subjects received the two interventions (ketamine and placebo). Thus, to build more compelling evidence, additional studies combining different technological approaches to the assessment of cognitive functions are required. Also, the effects of ketamine on cortical function along with other experimental and clinical conditions need to be evaluated.

Another critical issue is whether electrophysiological results can be translated into clinical effects. For example, whether an increase in BOLD induced by ketamine in neuroimaging studies can be translated into increased cortical activation. Hence, more extensive studies need to address these critical questions. Finally, it is also essential to understand the impact of ketamine in neuroplasticity—i.e., if ketamine can lead to long-term beneficial or harmful effects—and to understand the effects of ketamine in real clinical practice where patients are frequently taking several medications simultaneously.

## Conclusions and perspectives

In this review, we discussed the effects of ketamine on different ERP components mostly reflecting pre-attentive and attentional processes. It is necessary to point out that most studies exploring the effects of ketamine on ERPs were conducted to investigate the effects of ketamine in a model of glutamatergic dysfunction in healthy individuals. Despite the fact that many electrophysiological changes induced by ketamine correspond to findings in schizophrenic patients, ketamine does not produce a perfect model of schizophrenia in healthy subjects. Several ERP alterations were not reproduced by ketamine (such as sensory gating tests, and P1 and N1 components).

Overall, while some ERP changes following the intake of ketamine appear to reflect a decrease in the efficiency of most aspects of stimuli processing (reduced N2, P2, and P3 amplitudes, PN and MMN), other aspects were preserved or enhanced (P50 reduction, PPI, P1, and N1 amplitudes). It must be considered that the NMDA receptor is an ionotropic glutamatergic receptor which is widely distributed in the central nervous system from the dorsal horn of the spinal cord to the cortical areas. The NMDA receptor is implicated in several processes, exemplified here by information processing, learning and memory (Kocsis et al., 2013).

Additionally, regardless of ketamine being classified as an NMDA receptor antagonist, it can interact with a variety of receptors and channels, such as serotonin (Kapur and Seeman, 2002), opioid (Gupta et al., 2011), and dopamine (Seeman and Guan, 2008) receptors. Also, neuroimaging studies (Holcomb et al., 2001; Rogers et al., 2004; Deakin et al., 2008; Niesters et al., 2012) were not conclusive about the effect of ketamine in the brain cortex. Indeed, it has been shown that ketamine could increase levels of glutamate in specific areas (Holcomb et al., 2001; Deakin et al., 2008). Thus, it should be considered that the reduction or increase in ERP-amplitudes provoked by ketamine reflect complex processes and does not necessarily reflect cortical “hypoactivation” or “hyperactivation,” respectively, but that this can also indicate changes in baseline activation of many cortical areas.

The current findings suggest that ketamine may alter the perceived salience of different categories of stimuli. Thereby, this process might require changes in other cognitive resources to interpret the stimuli. For example, it has been demonstrated that the relationship between target and non-target (standard) stimuli strongly influences the P300 component (Polich, 2007), and that the processing of novel stimuli engages more cortical resources when overall task difficulty increases (Hagen et al., 2006). If ketamine alters the perceived relationship between the target and standard stimuli in the oddball task, it could potentially account for the P300 reductions and N100/P100 increases reported in most studies.

There is evidence of this effect of ketamine in many contexts involving discrimination between two categories of stimuli, such as auditory tones, geometrical images, self-produced and external vocalizations, and emotional and neutral faces. Interestingly, when used as an analgesic, a subanesthetic dosage of ketamine (0.3 mg/kg or less) is very effective in reducing pain unpleasantness, while perceived pain intensity does not seem to be greatly affected (Sigtermans et al., 2009). This fact suggests that the analgesic effect of ketamine may be attributed to a decreased affective discrimination of sensorial information (Sprenger et al., 2006). This concept is supported by neuroimaging studies which describe changes in the activity of specific brain areas related to the affective component of pain, such as the anterior cingulate cortex (ACC). Hence, this effect suggests that the euphoria provoked by ketamine actively interacts with the emotional aspect of pain more than with the sensorial aspect (Sprenger et al., 2006).

Most ERP components are subject to attentional modulation. Even though the attentional deficits produced by ketamine administration have been extensively studied, most results suggest that ketamine alters attentive function (as assessed by both specific attentional tasks and ERP studies). Only three studies did not found significant effects of ketamine on attention (Harborne et al., 1996; Adler et al., 1998; Newcomer et al., 1999). Therefore, ketamine effects should be interpreted along with reduced attentional levels.

Besides studies using ketamine as a model for schizophrenia, ERP findings have important implications for pain treatment as well as mood disorders and ketamine abuse. Future research may clarify ketamine's cortical effects in specific conditions.

## Author contributions

All authors approved the final manuscript. AS and WC: have searched the literature, reviewed the articles and written the text. FT and MZ: contributed to improve writing style and in bias assessment.

### Conflict of interest statement

The authors declare that the research was conducted in the absence of any commercial or financial relationships that could be construed as a potential conflict of interest.
